# Prolonged mechanical ventilation and its associated factors among adult patients admitted to intensive care unit in the teaching hospitals of Southern Ethiopia In 2023: a multicenter prospective cohort study

**DOI:** 10.1186/s12871-026-03660-y

**Published:** 2026-02-13

**Authors:** Tagesse Taye Dayama, Semagn Mekonnen Abate, Tajera Tageza Ilala, Bekele Buli Megersa, Yohannes Alemneh Alamirew

**Affiliations:** 1 Anesthesia Department, College of Medicine and Health Science, Hawassa University, Sidama, Ethiopia; 2 Anesthesia Department, College of Medicine and Health Science, Wollo University, Dassie, Ethiopia

**Keywords:** Intensive care unit, Mechanical ventilation, Prolonged mechanical ventilation, Critically ill patients, Risk factors, ICU outcomes

## Abstract

**Background:**

Prolonged mechanical ventilation (PMV) is defined as the ventilation of critically ill patients for ≥ 21 consecutive days for at least six hours per day. It has resulted in increased morbidity and mortality in patients admitted to the intensive care unit (ICU) in addition to huge hospital budget consumption. Identifying its risk factors may help to improve the intensive care unit outcomes and optimize resource allocation. Therefore, this study aimed to determine the incidence of prolonged mechanical ventilation and its associated factors among adult patients admitted to intensive care units in southern Ethiopia, 2023.

**Method:**

A multicenter prospective cohort study was conducted at ICUs in the teaching hospitals of southern Ethiopia among 390 adult patients mechanically ventilated for more than 24 h from February 2023 to October 2023.Binary logistic regression analysis was conducted to identify the factors associated with PMV. Multivariable logistic regression was fitted to determine the factors independently associated with PMV. In the final model, Adjusted Odds Ratio (AOR) and 95% confidence intervals (CI) were used to assess the strength of association and presence of statistical significance with a p-value of less than 5%.

**Results:**

Out of 390 patients included in this study, the incidence of PMV was 33.3% (CI 95%: 28.7–38.5). The odds of experiencing PMV were significantly lower among patients who underwent early tracheostomy compared with those who received late tracheostomy, with an adjusted odds ratio (AOR) of 0.08 (95% CI: 0.02–0.32; *p* = 0.001). The patients with anemia (AOR: 18.3 (95% CI: 3.54–60.27; *p* = 0.001)), at least one comorbidity (AOR: 39.9, 95% CI: 9.30-86.16; *p* = 0.0001), and parenteral nutrition (AOR: 9.8, 95% CI: 2.22–43.16; *p* = 0.003) were significantly associated with PMV.

**Conclusion:**

Overall, this study found a high incidence of prolonged mechanical ventilation (PMV). Anemia, early tracheostomy, the presence of at least one comorbidity, and parenteral nutrition were significantly associated with PMV which occurred more frequently among patients ventilated for respiratory causes. Therefore, optimization of overall ICU care, particularly hematologic management, and the application of targeted weaning strategies may help mitigate the burden and adverse outcomes of PMV.

## Introduction

Mechanical ventilation (MV) is the technique used to maintain adequate pulmonary gas exchange in critical patients due to various disease conditions in the intensive care unit ( ICU) [1]. The majority of patients require MV for a short time, whereas some require prolonged mechanical ventilation (PMV) (ventilation for more than 21 consecutive days) [2]. The incidence of PMV ranges from 6% to 28%, which is significantly associated with high psychological and economic impacts on both patients and their parents [3–5]. It also increases the burden on healthcare personnel and the consumption of ICU resources [3, 4].

PMV is associated with a number of factors, including not only patterns of ICU admission but also length of stay in the hospital before admission, high acute physiology and chronic health evaluation (APACHE) scores, the need for more vasoactive drugs, and the need for renal replacement therapy [6]. Numerous studies have shown that the patients with PMV are elderly and suffering from chronic obstructive lung disease (COPD), cancer, hypertension, diabetes mellitus, chronic heart failure, a low albumin level, and prolonged infusion of vasoactive agents [6–8].

In addition to the primary causes of ICU admission, complications that develop during follow-up are associated with PMV [6, 9]. Ventilator-associated pneumonia (VAP), acute respiratory distress syndrome (ARDS), acute kidney injury (AKI), pneumothorax, pulmonary embolism, and nosocomial infection are associated with PMV [10–12].

Globally, many studies have reported the prevalence, prognostic factors, and outcomes of PMV among patients admitted to ICUs [6, 13–15]. However, the evidence on the incidence and risk factors associated with PMV was lacking in sub-Saharan Africa as well as in Ethiopia.

Therefore, the objective of this study was to assess the incidence of prolonged mechanical ventilation (PMV) and its associated factors among adult patients on mechanical ventilation at ICUs in the teaching hospitals of southern Ethiopia, 2023.

## Methods and materials

### Study Area, Design, and period

This multicenter prospective cohort study was conducted at ICUs in the teaching hospitals of southern Ethiopia. Three teaching hospitals in southern Ethiopia were randomly selected by the lottery method, namely the Hawassa University Comprehensive Specialized Hospital (HUCSH), Wolayta Sodo Comprehensive Specialized Hospital (WSUCSH), and Dilla University Referral Hospital (DURH). The study was conducted from February 01, 2023, to October 30, 2023. Both ICUs have similar levels of care, staff profiles, ICU infrastructure, medical supplies, admission patterns, and monitoring modalities.

### Population

#### Source population

All adult (age ≥ 18 years) patients who were mechanically ventilated at ICUs in the teaching hospitals in southern Ethiopia.

#### Study population

All patients who were on invasive MV in the three randomly selected teaching hospitals in southern Ethiopia during the data collection period and who fulfill the inclusion criteria.

### Inclusion and exclusion criteria

#### Inclusion criteria

All adult patients admitted to the ICU who stay on invasive MV for more than 24 h were included in this study.

#### Exclusion criteria

Patients who were intubated and referred from other hospitals to the study area, patients who had repeated admissions and repeated intubation for the same illness, patients on MV prior to admissions, patients whose family/guardian refused, and those patients who required MV part-time or intermittently were excluded from the study.

### Variables of the study

#### Dependent variable

Prolonged mechanical ventilation.

#### Independent variables

Socio-demographics factors: Age, body mass index (BMI), and sex.

Types of ICU admission (medical, surgical, trauma, obstetrics and gynecology, and others (emergency and ear, nose, and throat (ENT))).

At least one comorbid illness (hypertension, diabetes mellitus, cardiac, respiratory, renal failure, neurological, and others (HIV/AIDS and cancers)).

Vital signs at ICU admission (heart rate (beats per minute), oxygen saturation (SpO₂, %), and respiratory rate (breaths per minute)).

ICU admission characteristics: Time of ICU admissions (day, night, weekend).

ICU interventions and services: ICU interventions such as corticosteroids, vasopressors or inotropes, parenteral nutrition, and tracheostomy.

Mechanical ventilation: modes of ventilation including volume-controlled ventilation (VCV), pressure-controlled ventilation (PCV), synchronized intermittent mandatory ventilation (SIMV), and pressure support ventilation (PSV); reason for the initiation of MV (respiratory, neurological, cardiovascular, traumatic, gastrointestinal, renal, and other (airway obstructions and organophosphate poisoning)). 

Complications during follow-up include ventilator-associated pneumonia (VAP), acute respiratory distress syndrome (ARDS), cardiac arrest, delirium, acute kidney injury (AKI), and others (pulmonary embolism, aspiration, and tube blockage).

Laboratory results including complete blood counts (hemoglobin, hematocrit, and platelet count); liver function tests (serum albumin level and total bilirubin); and renal function tests (serum creatinine (Cr) and blood urea nitrogen (BUN)).

Outcome: length of stay (LOS) in ICU.

### Sample size determination and sampling techniques

#### Sample size determination

**Fig. 1:**
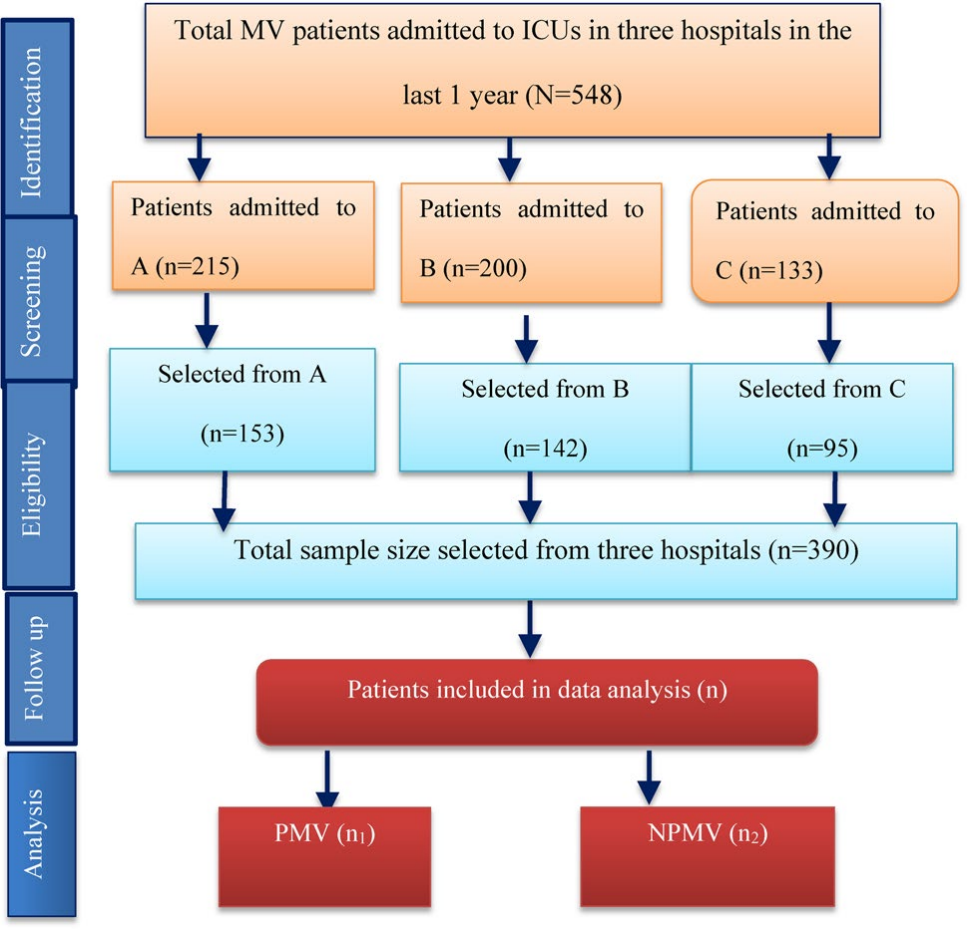
Strobe flow chart of patients admitted to ICUs in Southern Ethiopia

On the basis of a pilot study conducted at Wolayta Sodo Christian Hospital with 39 ICU patients, the sample size was determined via EpiInfo version 7.2.5.0. With a 95% confidence level, 80% power, proportions of 34.9% for PMV and 21% for NPMV, and a 2:1 ratio of non-exposed to exposed, the final sample size was calculated to be 390, comprising 130 patients with PMV and 260 patients with NPMV. According to the ICU logbooks from the three hospitals, the number of mechanically ventilated patients admitted over the previous 12 months was 215, 200, and 133 patients at HUCSH, WSUCSH, and DURH, respectively. To allocate the sample proportionally, the number of mechanically ventilated patients in each ICU was divided by the total number of ventilated patients across all sites and then multiplied by the total sample size (*n* = 390) (Fig. 1).

#### Sampling techniques

A consecutive sampling technique was used to include all adult MV patients who met the inclusion criteria and were admitted to the three selected hospitals from February 1, 2023, to October 30, 2023, until the required sample size was reached.

### Operational definitions

Invasive MV: The use of MV with endotracheal intubation or tracheostomy.

Prolonged mechanical ventilation: Patients who received MV for more than 21 consecutive days for at least six hours per day within 30 days of follow-up.

Parenteral Nutrition: the feeding of the patient intravenously, bypassing the gut.

Anemia: Hemoglobin level less than 10 mg/dl among patients admitted to ICUs.

### Data collection techniques and tools

Data were collected by using a structured questionnaire adapted from previous studies [6, 9, 16, 17] by kobo toolbox. Three senior anesthetists and three ICU critical care nurses who were not involved in patient management collected data prospectively after 24 h of ICU admissions for up to 30 days. The questionnaire comprises socio-demographic characteristics, including age, sex, and BMI. Admissions-related factors such as types of ICU admission and reasons for initiation of MV and modes of ventilation used, and vital signs at admission; complications during management such as ventilator-associated pneumonia (VAP), acute respiratory distress syndrome (ARDS), delirium, cardiac arrest, acute kidney injury (AKI), and others; interventions such as tracheostomy, parenteral nutrition, and vasopressor or inotropic use; comorbid conditions; laboratory results; duration of MV; and ICU length of stay (LOS). The follow-up time period was started at 24 h after ICU admission and continuously follows up to 30 days.

### Data quality control

Orientation was given for data collectors on the objectives of the study and contents of the questionnaire. Regular supervision and follow-up were done during data collection. Electronic data were checked for completeness and accuracy on a daily basis during data collection. Before data analysis, the data were coded, cleared, and checked for missing.

### Data management and analysis

Data were exported to SPSS version 26 for data processing, analysis, and performing statistical tests. Categorical data was presented by using frequency (percent). Normally distributed continuous data was presented by using mean and standard deviation. Non-normally distributed continuous data were reported by median and interquartile range (IQR). Histograms and Shapiro‒Wilk tests were used to check normality of distribution. Categorical covariates were analyzed by chi-squared statistics. Binary logistic regression was conducted to assess the factors associated with prolonged mechanical ventilation. Bivariable logistic regression analysis was done to select the candidate variables for multivariable logistic regression and to obtain crude association (Crude Odds Ratio (COR)). Variables with a p-value of less than 25% were inserted into the multivariable logistic regression models to determine the factors independently associated with prolonged mechanical ventilation and control confounders. Collinearity diagnostics among the independent covariates were checked by computing the linear regression models with the variance inflation factor (VIF) of less than 10 and tolerance test (> 0.1), which indicated there was no multicollinearity. Before fitting the final model, the model fitness was checked by the Hosmer–Lemeshow (HLS) goodness-of-fit test. The HLS goodness-of-fit test p-value is > 0.05, which indicates the model better fits the data. In the multivariable logistic regression models, AOR and 95% CI were used to determine the strength of association and presence of statistical significance at a p-value < 5%. The results were presented by using tables, graphs (bar graphs), and text. Finally, we reported the results of the present study in accordance with the Strengthening the Reporting of Observational Studies in Epidemiology (STROBE) guidelines for observational studies [18].

### Ethical considerations

An ethical clearance letter was received from Dilla University, Institutional Review Board (IRB), with the Ref. no. duirb/049/22 − 02, in accordance with the Helsinki Declaration [19]. A written permission letter was obtained from each teaching hospital’s administrative body and respective coordinators. Written informed consent was obtained from the guardians or close relatives before participation in the study because the patients themselves were incapacitated and unable to provide consent. All the information and identities of the patients were kept confidential.

## Results

### Socio-demographics and admission characteristics of adult patients admitted to the ICU in Southern Ethiopia

A total of 390 adult patients were included in this study, with a response rate of 100%. The majority of patients who required prolonged mechanical ventilation (PMV) were males aged between 18 and 29 years. Approximately 66.2% of PMV patients had a GCS score less than 8. Most PMV patients were admitted to the ICU from the medical ward (61.5%), followed by the emergency trauma unit (16.2%), the surgical ward (9.2%), other departments (6.9%), and obstetrics and gynecology (6.2%). The primary reason for the initiation of MV was respiratory disorders (25.4%), followed by neurologic (23.8%) and trauma-related disorders (13.8%). However, the reason for the initiation of MV was the lowest compared to others (8.5%) (Table 1).

**Table 1:** Baseline sociodemographic and admission characteristics of adult patients admitted to the ICU in Southern Ethiopia, 2023 (*n* = 390)

Variables	Category	ICU admissions (%)	Prolonged MV, frequency (%)		P-value
			No = 260(66.6)	Yes = 130(33.3)	
Sex	Female	151(38.7)	94(35.8)	58(44.6)	1
	Male	239(61.3)	167(64.2)	72(55.4)	0.27
Age (years)	18–29	149(38.2)	98(37.7)	51(38.2)	0.65
	30–49	182(46.7)	127(48.8)	55(42.3)	0.41
	≥ 50	59(15.1)	35(13.5)	24(18.5)	0.59
BMI (kg/m^2^)	< 30	370(94.9)	253(97.3)	117(90.0)	1
	≥ 30	20(5.1)	7(2.7)	13(10.0)	0.004*
GCS scores	≤ 8	219(56.2)	133(51.2)	86(66.2)	0.005*
	>8	171(43.8)	127(48.8)	44(33.8)	1
Time of ICU admissions	Day	214(54.9)	141(54.2)	73(56.2)	0.81
	Night	113(29.0)	77(26.6)	36(27.7)	0.67
	Weekend	63(16.2)	42(16.2)	21(16.2)	0.919
Types of ICU admissions	Medical	215(55.1)	135(51.9)	80(61.5)	0.991
	Surgical	33(8.5)	21(8.1)	12(9.2)	0.441
	Trauma	67(17.2)	46(17.7)	21(16.2)	0.651
	Obsterics and gynacology	32(8.2)	24(9.2)	8(6.2)	0.771
	Others	43(11.0)	34(13.1)	9(6.9)	0.296
Reasons for intiation MV at admissions	Respiratory	101(25.9)	68(26.2)	33(25.4)	0.401
	Neurological	63(16.2)	32(12.3)	31(23.8)	0.593
	Cardiovascular	35(9.0)	25(9.6)	10(7.7)	0.253
	Truama	62(15.9)	44(16.9)	18(13.8)	0.560
	Gastro intestinal	54(13.8)	39(15.0)	15(11.5)	0.906
	Renal	44(11.3)	32(12.3)	12(9.2)	0.273
	Others	31(7.9)	20(7.7)	11(8.5)	0.616

Types of ICU admissions; others (emergencies and ENT). Reasons for initiation of MV at admissions: others (postoperative cases and organophosphate poisoning). *GCS* Glasgow Coma Scale, *BMI* Body Mass Index

### Vital signs, laboratory results, and comorbidities at admission among patients admitted to the ICU in Southern Ethiopia

More than 60% of patients who required prolonged mechanical ventilation had oxygen saturation above 90%, a respiratory rate greater than 20 breaths per minute, and a heart rate of at least 100 beats per minute. However, none of these variables showed a statistically significant association with PMV (*P* > 0.05).

More than half of the patients with prolonged mechanical ventilation had serum albumin levels below 2.5 g/dL, total bilirubin ≥ 1.2 mg/dL, platelet counts < 150 × 10³/µL, creatinine ≥ 2 mg/dL, and blood urea nitrogen ≤ 25 mg/dL. However, only a low platelet count was significantly associated with prolonged mechanical ventilation (*P* < 0.005) (Table 2).

**Table 2:** Vital signs, laboratory results, and comorbidities at admission among patients admitted to the ICU in the Southern Ethiopia, 2023 (*n* = 390)

Variables	Category	ICU admissions(*n* = 390)	Incidence of prolonged MV, frequency (%)		*P*-value
			No = 260 (66.7)	Yes = 130 (33.3)	
SPO2(%)	≥90	268(68.7)	189(72.7)	79(60.8)	1
	< 90	122(31.3)	71(27.3)	51(39.2)	0.37
Respiratory rate	≤ 12	8(2.1)	3(1.2)	5(3.8)	0.95
	12–20	117(30.0)	75(28.8)	42(32.3)	0.88
	≥20	265(67.9)	182(70.0)	83(63.8)	0.49
Heart rate	≤60	14(3.6)	10(3.8)	4(3.1)	0.56
	60–100	139(35.6)	96(36.9)	43(33.1)	0.98
	≥ 100	237(60.8)	154(59.2)	83(63.8)	0.45
MAP(mmHg)	Mean±SD	82.04±13.8	82.3±13.4	83.3±14.6	0.506
Hematocrit(%)	< 30	103(26.4)	63(24.2)	40(30.8)	0.268
	≥ 30	287(73.6)	197(75.8)	90(69.2)	1
Hemoglobin (g/dL)	< 10	136(34.9)	86 (33.1)	50(38.5)	0.223
	≥ 10	254(65.1)	174(66.9)	80(61.5)	1
Platelets count/µL	≥ 150 × 10^3^	226(57.9)	172(66.2)	54(41.5)	1
	< 150 × 10^3^	164(42.1)	88(33.8)	76(58.5)	0.0001*
Albumin Levels (g/dL)	> 2.5	106(27.2)	80(30.8)	26(20.0)	1
	≤ 2.5	284(72.8)	180(69.2)	104(80)	0.025*
Total bilirubin (mg/dL)	< 1.2	218(55.9)	136(52.3)	82(63.1)	1
	≥ 1.2	172(44.1)	124(47.7)	48(36.9)	0.562
Creatinine (mg/dL )	< 2	203(52.1)	150(57.7)	53(40.8)	1
	≥ 2	187(47.9)	110(42.3)	77(59.2)	0.002*
Blood urea nitrogen(mg/dL)	≤ 25	249(63.8)	178(68.5)	71(54.6)	1
	> 25	141(36.2)	82(31.5)	59(45.4)	0.008*
At least one comorbidity	No	265(67.9)	220(84.6)	45(34.6)	
	Yes	125(32.1)	40(15.4)	85(65.4)	0.0001*

*Statistically significant; SPO2: peripheral oxygen saturation. Comorbidities: others (HIV/ADIS and Cancers)

### Ventilator modes, and settings, interventions and complications of adult patients admitted to the ICU in Southern Ethiopia

Among the interventions assessed, the majority of PMV patients received parenteral nutrition (43.8% vs. 13.1%), corticosteroid therapy (45.4% vs. 20.4%), and vasopressors or inotropes (42.3% vs. 16.5%), which were greater percentages than those receiving NPMV. However, tracheostomies performed were nearly equal between two groups (25.4% vs. 26.9%).

The most predominant initial mode of ventilation was volume control, and the least frequent was pressure support ventilation in patients with PMV. More than 60% of the PMV patients had a mean tidal volume (TV) > 6 mL/kg, positive end expiratory pressure (PEEP) > 5 cmH₂O, and fraction of inspired oxygen (FiO₂) > 0.6 within the first 72 h of ICU admission.

In the ICU, each patient had developed at least one complication. The most complications developed in patients with PMV were ventilator-associated pneumonia (VAP) (18.5%) and acute respiratory distress syndrome (ARDS) (18.5%). However, the least was Acute Kidney Injury (AKI) (6.9%). The median length of ICU stay was 24 days (IQR: 21–30) among those patients with PMV as compared to NPMV, 14 days (IQR: 2–19) (Table 3).

**Table 3:** ICU interventions among adult patients admitted to the ICU in the Southern Ethiopia, 2023 (*n* = 390)

Variables	Category	ICU admissions, frequency (%)	Prolonged MV, frequency (%)		*P* value
			No = 260(66.7)	Yes = 130(33.3)	
Mode of ventilation	VCV	251(64.4)	163(62.7)	88(67.7)	>0.05
	PCV	47(12.1)	33(12.7)	14(10.8)	
	SIMV	72(18.5)	50(19.2)	22(16.9)	
	PSV	20(5.1)	14(5.4)	6(4.6)	
Tidal volume	≤ 6 mL/kg	121(31.0)	85(32.7)	36(27.7)	1
	> 6 mL/kg	269(69.0)	175(67.3)	94(72.3)	0.314
PEEP(cmH₂O)	≤ 5	114(29.2)	67(25.8)	47(36.2)	1
	> 5	276(70.8)	193(74.2)	83(63.8)	0.278
FiO₂	≤ 0.6	65(16.7)	43(66.2)	22(16.9)	1
	> 0.6	325(83.3)	217(83.5)	108(83.1)	0.923
Plateau pressure(cmH₂O)	≤ 30	342(87.7)	230(88.5)	112(86.2)	0.513
	> 30	48(12.3)	30(11.5)	18(13.8)	1
Tracheostomy	No	287(73.6)	290(73.1)	97(74.6)	1
	Yes	103(26.4)	70(26.9)	33(25.4)	0.745
Duration of tacheostomy	≤ 10days	76(62.8)	62(79.5)	14(32.6)	0.0001*
	> 10 days	45(37.2)	16(20.5)	29(67.4)	1
Vasopresor (Inotropes)	No	292(74.9)	217(83.5)	75(57.7)	1
	Yes	98(25.1)	43(16.5)	55(42.3)	0.0001*
Parentral nutrition	No	299(76.7)	226(86.9)	73(56.2)	1
	Yes	91(23.3)	34(13.1)	57(43.8)	0.0001*
Use of corticosteroids	No	291(74.6)	207(79.6)	84(64.6)	1
	Yes	99(25.4)	53(20.4)	46(45.4)	0.001*
ICU complications	VAP	77(19.7)	53(20.4)	24(18.5)	0.694
	ARDS	74(19.0)	40(15.4)	24(18.5)	0.283
	Cardiac arrest	56(14.4)	37(14.2)	19(14.6)	0.255
	Delirum	53(13.6)	36(13.8)	17(13.1)	0.256
	AKI	27(6.9)	18(6.9)	9(6.9)	0.442
	Others	39(10.0)	28(10.8)	11(8.5)	0.344
LOS in ICU (days)		8 (2–30)	14(2–19)	24 (21–30)	

*Statistical significant. *VCV* Volume controlled ventilation, *PCV* Pressure control ventilation, *SIMV* Synchronized intermittent mandatory ventilation, *PSV* Pressure support ventilation, *VAP* Ventilator-associated pneumonia, *LOS* Length of Stay, *IQR* Inter Quartile Range

### Incidences of PMV among adult patients admitted to ICUs in Southern Ethiopia

**Fig. 2:**
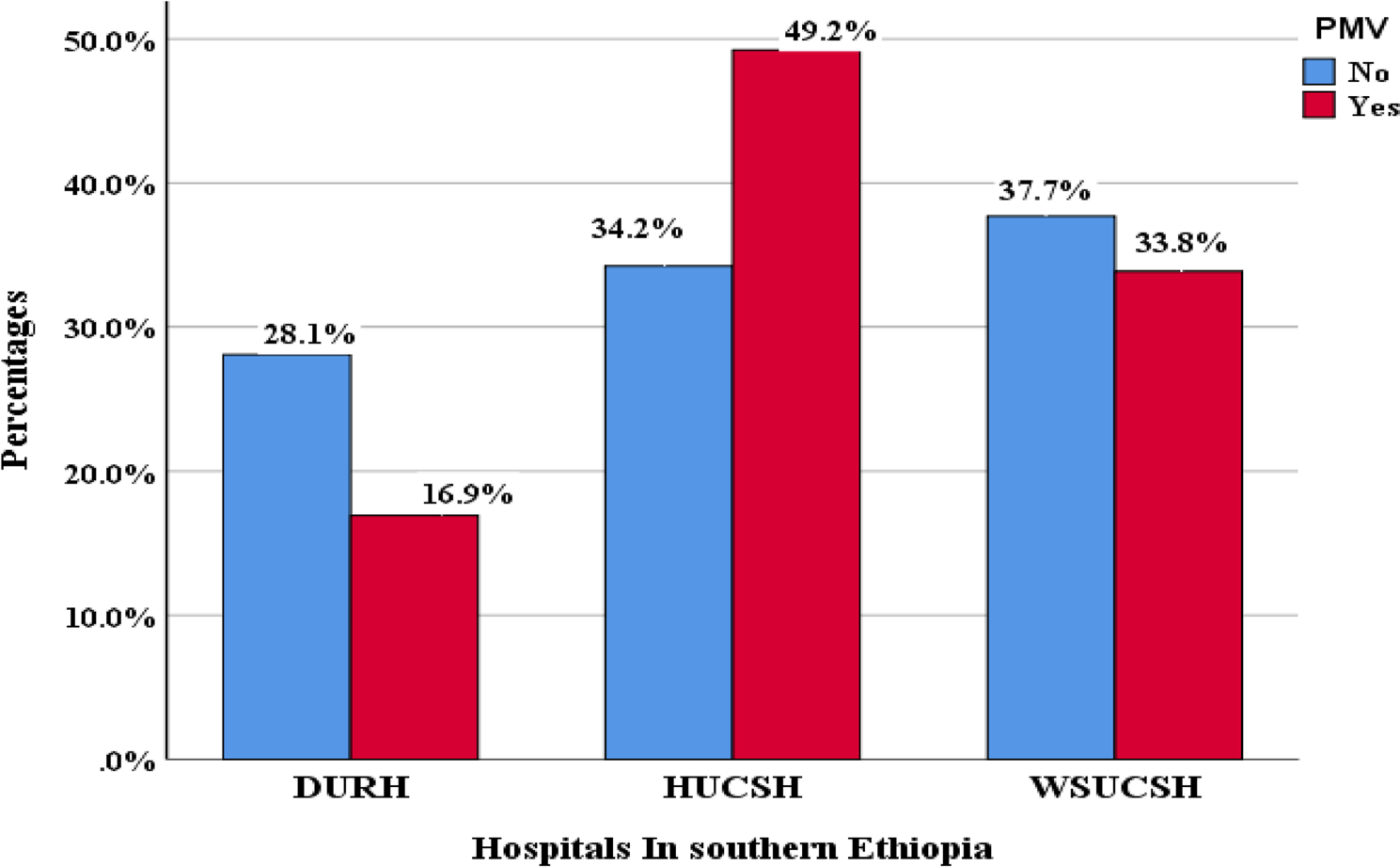
Overall incidence of PMV among adult patients in ICUs Southern Ethiopia

The incidence of PMV among patients admitted to ICUs in southern Ethiopia was 33.3% (95% CI: 28.7–38.5), obtained from 1000 bootstrap resamples. It was highest at Hawassa University Comprehensive Specialized Hospital (HUCSH) at 49.2%, followed by Wolaita Sodo Comprehensive Specialized Hospital (WSCSH) at 33.8%, and lowest at Dilla University Referral Hospital (DURH) at 16.99% (Fig. 2).

### Factors associated with PMV among adult patients admitted to the ICU in Southern Ethiopia

This prospective multicenter cohort study aimed to identify independent predictors of PMV. For bivariate analysis, variables with a p-value less than 0.25 were entered into multivariate analysis via the forward Wald selection method. Model fitness was evaluated with Pearson chi-square statistics (X² = 83.727, df = 5, *p* = 0.0001), pseudo R² = 0.686, and the insignificant Hosmer–Lemeshow test (X² = 12.195, df = 8, and *p* = 0.143), and 68.6% of the variation in PMV was explained by the independent variables included in the final regression model. The remaining 31.4% were due to other causes.

Anemia, at least one comorbidity, tracheostomy duration, and parenteral nutrition, according to multivariate analysis, were significantly associated with PMV. The multivariate analysis revealed that patients with anemia were approximately eighteen times more likely to be on PMV than were those who were normal, with an AOR of 18.3 (95% CI: 3.54–60.27; *p* = 0.001). Furthermore, the multivariate analysis revealed that patients who took parenteral nutrition during follow-up were approximately ten times more likely to be on PMV than those who did not (AOR: 9.8, 95% CI: 2.22–43.16; *p* = 0.003) (Table 4).

**Table 4:** Factors associated with PMV among adult patients admitted to the ICU in Southern Ethiopia, 2023 (*n* = 390)

Variables	Category	Prolonged MV, frequency (%)		COR(95% CI)	P value	AOR(95%CI)	P value
		No = 260	Yes = 130				
BMI (kg/m^2^)	< 30	253(97.3)	117(90.0)		1		
	≥ 30	7(2.7)	13(10.0)	4.0(1.56–10.33)	0.004*	0.1(0.01–1.10)	0.58
GCS scores	≤ 8	133(51.2)	86(66.2)	1.9(1.21–2.89)	0.005*		
	>8	127(48.8)	44(33.8)		1		
Hemoglobin (g/dL)	< 10	86 (33.1)	50(38.5)	1.3(0.82–1.96)	0.223	**18.3(3.54–60.27)**	0.001*
	≥ 10	174(66.9)	80(61.5)		1		1
Platelets count/µL	≥150 × 10^3^	172(66.2)	54(41.5)				
	< 150 × 10^3^	88(33.8)	76(58.5)	2.8(1.88–4.24)	0.0001*	1.5(0.67–9.01)	0.053
Albumin Levels (g/dL)	>2.5	80(30.8)	26(20.0)				
	≤ 2.5	180(69.2)	104(80)	1.8(1.07–2.94)	0.025*		
Creatinine (mg/dL )	< 2	150(57.7)	53(40.8)		1		
	≥ 2	110(42.3)	77(59.2)	2.0(1.29–3.04)	0.002*		
Blood urea nitrogen(mg/dL)	≤ 25	178(68.5)	71(54.6)				
	> 25	82(31.5)	59(45.4)	1.8(1.17–2.78)	0.008		
Vasopresor (Inotropes)	No	217(83.5)	75(57.7)		1		
	Yes	43(16.5)	55(42.3)	3.7(2.30–5.97)	0.0001*		
Parentral nutrition	No	226(86.9)	73(56.2)				1
	Yes	34(13.1)	57(43.8)	5.2(3.15–8.56)	0.0001*	**9.8(2.22–43.16)**	0.003*
Atleast one comorbidity	No	220(84.6)	45(34.6)		1		
	Yes	40(15.4)	85(65.4)	10.4(6.34–17.03)	0.0001*	**39.9(9.30-86.16)**	0.0001*
Corticosteroids	No	207(79.6)	84(64.6)				
	Yes	53(20.4)	46(45.4)	2.1(1.34–3.42)	0.001*		
Duration of tacheostomy	≤ 10days	62(79.5)	14(32.6)	0.13(0.05–0.29)	0.0001*	**0.08(0.02–0.32)**	0.001*
	> 10 days	16(20.5)	29(67.4)				**1**

*Statistically significant. *BMI* Body mass index

Bold data indicate statistically significant results (p < 0.05), with 95% confidence intervals not crossing 1

## Discussions

This is a multicenter prospective cohort study, which was conducted to investigate the incidence of PMV and its associated factors among adult patients admitted to intensive care units in southern Ethiopia. Our study summarizes the pattern-related and intervention-related factors that may affect the duration of MV. In the current study parenteral nutrition, anemia, early tracheostomy, and at least one comorbidity were identified as independent associated factors with PMV.

The majority of the patients with PMV admitted to the ICU in this study were younger, with a median age of 34 (range: 18–80) years. This median age is particularly lower than that reported in subsequent studies [20–23]. The younger age group might be more involved in trauma-related events such as war, violence, and road traffic accidents and relatively engaged in high-risk behaviors or occupations, which could explain the variation in this age group in developing countries, unlike in developed countries, where older patients dominate ICUs due to comorbidities.

The incidence of PMV in this study was 33.3%, which was higher than that reported in the previous studies [2, 4, 6, 14, 15]. The reason for these variations might be related to several contextual and system-level factors, such as many patients being referred to our ICUs from a wide catchment area, late referrals to our ICUs with advanced diseases and multi-organ dysfunctions, and lack of specialized units, weaning protocols, hemodialysis, and delayed interventional procedures.

The majority of patients with PMV in this study were from medical wards, which is consistent with research from Taiwan [24] and Brazil [6, 25]. However, the majority of these patients were from the emergency department in Japan [20] and the general ward in Singapore [22]. These variations might be due to variations in ICU specialties and referral systems.

The main reason for initiation of MV in PMV patients in this study was respiratory disorders, which is in line with studies conducted in Western [6, 25, 26] and Asian countries [7, 15, 24, 27, 28]. In contrast, cardiovascular disorders were the main reason in the studies conducted in France [29], sepsis in Turkey [9],and trauma in Canada [30]. The reason for this discrepancy could be the availability of specialized units and disease prevalence.

Patients with at least one comorbidity were significantly associated with PMV in this study, which aligned with the previous studies [31–33].However, in other studies comorbidities were not significantly associated with PMV [34, 35]. The reason might be due to differences in study populations, types and severity of comorbid conditions, and variations in the statistical adjustment for acute illness severity.

In our study patients with early tracheostomy significantly decreased the risk of developing prolonged MV duration, which lined up with a study conducted by Merola et al. [36].This finding suggests that early tracheostomy might facilitate early weaning and decrease the duration of MV.However, another study conducted by Liu et al. reported that the duration of MV has no significant association between early and tracheostomy [32]. This might be due to differences in study populations, definitions of “early” and “late” tracheostomy, heterogeneity in illness severity, and variations in ICU practices and ventilation weaning protocols across studies.

In this study patients with thrombocytopenia in bivariate analysis have significantly associated prolonged MV. However, in the multivariate analysis, thrombocytopenic patients had no significant association with PMV, which contradicted the following study conducted by Li et al. [37]. This might be associated with underlying disease conditions such as sepsis, septic shock, or multiple organ dysfunctions.

In this study, parenteral nutrition was found as a significantly associated factor of PMV. This finding is consistent with the following findings [38, 39]. In contrast, a systematic review and meta-analysis reported that there was no association between parenteral nutrition and duration of MV [40]. The reason for this discrepancy might be explained by differences in patient populations, where patients receiving parenteral nutrition are often more critically ill or have gastrointestinal dysfunction; differences in timing and composition of nutritional support; and variations in ICU practices and weaning protocols.

In some studies anemia was significantly associated with PMV, which is in line with the following studies [41, 42]. However, a study conducted by Lia et al. reported that anemia was not an independent risk factor associated with PMV [43]. The reason might be associated with differences in study populations, definitions of anemia, illness severity, and variations in transfusion thresholds or clinical practices across studies.

The strength of this study is that it is the first multicenter prospective cohort study conducted in Ethiopia to explore the incidence and predictors of PMV among patients admitted to mixed ICUs. However, this study has certain limitations. First, disease severity scores could not be calculated because of the unavailability of laboratory parameters. Second, the study populations were heterogeneous, including medical, surgical, trauma, obstetric, and gynecological, which may affect the observed associations. Lastly, specific subgroup analyses were not conducted due to the small sample size of pathological categories to better delineate differential risks of PMV. Therefore, future studies with larger sample sizes and multicenter designs should perform pathology-specific subgroup analyses to better delineate differential risks for PMV.

Various clinical factors like anemia, at least one comorbidity, early tracheostomy, and parenteral nutrition were significantly associated with PMV. These findings highlight the importance of early risk identification and close monitoring of high-risk patients by trained ICU professionals, with particular attention to optimization of clinical management and implementation of standardized weaning protocols. We recommend that the Ethiopian Ministry of Health strengthen critical care capacity by providing targeted training for healthcare professionals and expanding multidisciplinary ICU services. Furthermore, future researchers should conduct prospective longitudinal studies with larger cohorts to evaluate both in-ICU and post-discharge outcomes of patients requiring prolonged mechanical ventilation.

## Conclusions

Overall, this study found a high incidence of prolonged mechanical ventilation (PMV). Anemia, early tracheostomy, the presence of at least one comorbidity, and parenteral nutrition were significantly associated with PMV. These factors may serve as indicators for identifying patients at increased risk of prolonged ventilatory support. PMV occurred more frequently among patients ventilated for respiratory causes. Optimization of overall ICU care, particularly hematologic management, and the application of targeted weaning strategies may help mitigate the burden and adverse outcomes of PMV.

## Data Availability

the datasets are not publicly available owing to ethical issues and confidentiality but are available upon reasonable request by the corresponding author.

## Abbreviations

PMV: Prolonged mechanical ventilation
NPMV: Non-prolonged mechanical ventilation
MV: Mechanical ventilation
AKI: Acute kidney injury
AORs: Adjusted odd ratios
BMI: Body mass index
GCS: Glasgow Coma Scale
ICU: Intensive care unit
LOS: Length of stay
VAP: Ventilator-associated pneumonia
